# Savanna chimpanzee tool and fruit transfers facilitate the transmission of technological behaviours at Dindefelo, Senegal

**DOI:** 10.3389/fpsyg.2026.1868894

**Published:** 2026-09-16

**Authors:** Andreu Sánchez-Megías, Amanda Barciela, Kevin López-Leal, Flavia Piccolella, Carlota F. Galán-Plana, Nadia Mirghani, Manuel Llana, Jordi Galbany, Laia Dotras, Justinn Renelies-Hamilton, Adrián Arroyo, R. Adriana Hernandez-Aguilar

**Affiliations:** 1 Department of Social Psychology and Quantitative Psychology, Faculty of Psychology, University of Barcelona, Barcelona, Spain; 2 Jane Goodall Institute Spain in Senegal, Dindefelo Biological Station, Dindefelo, Kedougou, Senegal; 3 Department of Primate Behaviour and Evolution, Max Planck Institute for Evolutionary Anthropology, Leipzig, Germany; 4 Department of Clinical Psychology and Psychobiology, Faculty of Psychology, University of Barcelona, Barcelona, Spain; 5 Institute of Neurosciences, University of Barcelona, Barcelona, Spain; 6 Seminar on Prehistoric Studies and Research (SERP), University of Barcelona, Barcelona, Spain; 7 Institute of Archaeology of the University of Barcelona (IAUB), Barcelona, Spain; 8 Serra Hunter Programme, Generalitat de Catalunya, Spain

**Keywords:** material culture, perishable tools, proto-tool use, scaffolding, tool use

## Abstract

Social learning is a key factor in the transmission of technological behaviours amongst primates. We studied chimpanzee (*Pan troglodytes verus*) social interactions in the context of tool and proto-tool use behaviours in the savanna site of Dindefelo, Senegal. Between 2017 and 2025, we obtained 14 h 58 min of camera trap footage at 14 locations where chimpanzees performed tool-use behaviours, namely algae fishing (for filamentous green algae: Zygnemataceae), ant dipping (for army ants: *Dorylus* sp.), and termite fishing (for termites: Macrotermitinae). We also obtained 30 min of camera trap footage at 11 locations where chimpanzees carried baobab (*Adansonia digitata*) fruit, which they pound against stationary anvils (i.e., proto-tools), a behaviour called baobab pounding. In total, we recorded 33 tool transfers and four fruit transfers. Transfers occurred mostly between mothers and their offspring. Most tool transfers were recoveries and tolerated takes. We compared the behaviour of initial tool possessors and tool recipients 30 s after and 30 s before each transfer. We excluded recoveries because in these cases the initial possessor does not interact with the recipient before abandoning the tool. We fitted Linear Mixed-Effects Models to test whether initial possessors and recipients exhibited different proportions of time using tools, insertions per second, and mouthfuls per second, after versus before a transfer. Initial possessors spent a significantly lower proportion of time using tools and performed significantly fewer insertions per second after transferring a tool, whereas recipients spent a significantly higher proportion of time using tools, and performed significantly more insertions and mouthfuls per second after receiving a tool. In addition, we detail two cases that highlight social learning of technological behaviours, in the context of ant dipping and baobab pounding, respectively. In line with previous research, we found that tool transfer constitutes a type of teaching in Dindefelo. Our results support the idea that teaching and facilitation, mainly from mothers, contribute to the acquisition of technological skills in immature chimpanzees.

## Introduction

Social learning allows animals to acquire knowledge from others, which can lead to the emergence of group-specific traditions and cultures (Thornton and Clutton-Brock, 2011). This process plays an important role throughout the lives of primates. Primates first learn from their caregivers, which in most species are primarily the mothers, then also from an increasing number of conspecifics (Whiten and van de Waal, 2018). In addition, migrating adults learn from the group’s resident individuals to adapt to the social and ecological conditions of their new environment (Luncz and Boesch, 2014; Whiten and van de Waal, 2018). Amongst the most challenging behaviours to acquire for non-human primates are those involving tool use (Boesch et al., 2019). Mounting evidence supports that social learning is key in the acquisition of such behaviours (Whiten, 2021), particularly during infancy, which represents a critical period to acquire certain tool-use behaviours (Biro et al., 2003). Young individuals recognise skilled conspecifics as models, whom they observe and copy, in a master-apprentice relationship (Matsuzawa et al., 2001). Individuals alternate observation with practise, which has been suggested to progressively increase the expertise of the learner until fully mastering complex tool-use behaviours (Whiten, 2015).

Learning through observing and copying others has been thought to be motivated by the learner’s desire to obtain a reward, yet multiple examples of primate traditions without a clear reward support instead that it may be motivated by the learner’s internal drive to copy others and fit in (de Waal, 2001). In this context, the learner’s success in acquiring a new behaviour would depend on identifying the model, the model’s tolerance with the observer, and the social relationship between them (de Waal, 2001). Primates are known to spontaneously match the actions of a model without receiving a reward (Fuhrmann et al., 2014; Yu et al., 2018). Their mirror neurons fire not only when performing an action, but also when observing it or hearing its sound (Kohler et al., 2002; Le Bel et al., 2009; Huang et al., 2019; Huang and Brosch, 2020). Primates are species whose survival depends partly on their social coordination (Yu et al., 2018), which could explain their tendency to copy others (de Waal and Bonnie, 2009). In chimpanzees (*Pan troglodytes*), mirror neurons fire when exposed to actions directed to objects (transitive) and also to actions not directed to objects (intransitive), same as in humans (Hetch et al., 2012; Hetch et al., 2013). In contrast, Rhesus macaque (*Macaca mulatta*) mirror neurons activate only in response to object-directed actions (Hetch et al., 2012). In addition, the differences found in the mirror neuron networks amongst these species may explain why chimpanzees and humans are able to copy and imitate fine motor movements, which may be linked to their social learning abilities of tool-use behaviours (Hetch et al., 2012; Hetch et al., 2013).

When the model takes an active role and modifies their behaviour to actively facilitate the observer’s learning, it is considered teaching (Hoppitt et al., 2008). To be classified as such, teaching must occur in the learner’s presence, produce a cost or no benefit to the teacher, and facilitate the naïve individual’s learning (Caro and Hauser, 1992). Additional criteria for teaching include that the model understands the learner’s ability and evaluation, and that they produce ostensive signals (Musgrave et al., 2016). This cooperative behaviour requires teacher-learner coordination, and its success depends on the teacher’s effectiveness and the learner’s ability (Thornton and Raihani, 2008). In addition to humans, there is evidence of teaching in social insects, birds, meerkats, felids, and other primates (Hoppitt et al., 2008; Fragaszy et al., 2013; Musgrave et al., 2016). This suggests that multiple taxa have evolved teaching to transmit complex information (Thornton and Raihani, 2008). Teaching may emerge when it is the optimal strategy to transfer intricate information (Musgrave et al., 2016). This idea is supported by evidence of non-human primate teaching in the context of motor skill development (Maestripieri, 1995; Whiten, 1999) and foraging behaviour (Humle and Snowdon, 2008; Rapaport and Brown, 2008), including tool use (Musgrave et al., 2016).

Chimpanzees have the most diverse array of technological behaviours of all non-human animals (Whiten et al., 1999; Kühl et al., 2019) and social learning is known to facilitate the acquisition of their tool-use behaviours (Humle et al., 2009; Fragaszy et al., 2013; Whiten, 2021). Naïve individuals learn mainly by observing others or by interacting with the remnants of the activities of more skilled individuals (Biro et al., 2003; Hopper et al., 2015). Chimpanzees learn more successfully after directly seeing the actions of others, highlighting the importance of observational learning (Hopper et al., 2015). In addition, chimpanzees transfer tools between individuals, which has been documented in the context of multiple tool-use behaviours: nut cracking (*Coula edulis* and *Panda oleosa*: Boesch, 1991), termite fishing (Isoptera: Lonsdorf, 2006; Pruetz and Lindshield, 2012; Sanz and Morgan, 2013; Musgrave et al., 2016), ant fishing for arboreal species (*Camponotus* spp. and *Crematogaster* spp.: Nishida and Hiraiwa, 1982), ant dipping for terrestrial species (*Dorylus burmeisteri*: Pruetz and Lindshield, 2012) and galago hunting (*Galago senegalensis*: Pruetz and Lindshield, 2012). Tool transfer happens mostly from adults to infants, especially from mothers to offspring (Lonsdorf, 2006; Musgrave et al., 2016). Tool transfer from more proficient to less experienced individuals is considered a form of teaching since it fulfils the criteria for teaching listed above (Musgrave et al., 2016).

For infant chimpanzees, their mothers represent the most important social facilitators, models and teachers (Whiten and van de Waal, 2018). Mothers facilitate their infants’ learning by providing tool-use opportunities, encouraging their tool-use attempts, reducing tool-use associated risks, providing tools, and acting as observational models (Boesch, 1991; Biro et al., 2003; Humle et al., 2009; Fragaszy et al., 2013). Tool transfer and other forms of teaching occur in chimpanzees, suggesting that mothers more actively facilitate the transmission of complex behaviours, such as those involving tool manufacturing or tool-set use (Sanz et al., 2004; Musgrave et al., 2020). Thus, teaching, including tool transfer, may contribute to the transmission of complex material culture in chimpanzees (Musgrave et al., 2020). Through social learning, chimpanzees acquire skills that they cannot innovate on their own (van Leeuwen et al., 2024). In addition, an individual’s culture influences how they solve new foraging problems (Gruber et al., 2009). Despite this evidence, how widespread teaching is amongst chimpanzees across Africa and the role it plays in different technological behaviours remain poorly known (Musgrave et al., 2020).

Here, we aimed to study the role of social learning in the acquisition of technological behaviours amongst savanna chimpanzees of Dindefelo, Senegal. Dindefelo chimpanzees are known to exhibit four technological behaviours for foraging. They use stick tools for three purposes, gathering filamentous algae (*Mougeotia* spp. and *Spirogyra* spp.: Jane Goodall Institute Spain, unpublished data) from ponds (algae fishing: Kühl et al., 2019), army ants (*Dorylus* sp.) from subterranean ant nests or surface trails (ant dipping: Sánchez-Megías et al., 2024, 2026), and termites (*Macrotermes* spp. and *Odontotermes* spp.: Jane Goodall Institute Spain, unpublished data) from termite mounds (termite fishing: Boesch et al., 2020). Moreover, Dindefelo chimpanzees use fixed stone or wooden anvils (also called passive hammers: Harmand and Arroyo, 2023) against which they crack open baobab (*Adansonia digitata*) fruits (baobab pounding: Galán-Plana et al., 2026). The latter is classified as a proto-tool-use behaviour given that the chimpanzees do not manipulate or modify the anvils (sensu Parker and Gibson, 1977; Shumaker et al., 2011; Jacobs and Osvath, 2016). Termite fishing and ant dipping occur in chimpanzee communities across Africa (Schöning et al., 2008; Sanz and Morgan, 2013; Kühl et al., 2019; Boesch et al., 2020). In contrast, algae fishing and baobab pounding are behaviours restricted to West African chimpanzees (*P. t. verus*) (Baldwin, 1979; McGrew et al., 2003; Marchant and McGrew, 2005; Duvall, 2008; Boesch et al., 2017; Kühl et al., 2019; Pruetz et al., 2020; Lindshield et al., 2021a).

Similar to what has been reported for termite fishing in Goualougo, the Republic of the Congo (Musgrave et al., 2016), we hypothesised that tool transfers in Dindefelo would represent a form of teaching of tool-use behaviours and, therefore, they would represent a cost to the initial possessor and a benefit to the recipient. We studied the behaviour of tool initial possessors and recipients during the moments immediately before and after tool transfer in the context of algae fishing, ant dipping and termite fishing. We predicted that tool initial possessors would exhibit a lower proportion of time using tools, tool insertions per second, and mouthfuls per second, and that tool recipients would exhibit a higher proportion of time using tools, tool insertions per second, and mouthfuls per second after a tool transfer compared to before (following Musgrave et al., 2016). We also studied instances of baobab fruit transfer. Finally, we provide detailed descriptions of two cases (one of ant dipping and one of baobab pounding) that highlight the social learning aspects involved in the transmission of technological behaviours by these chimpanzees.

## Methods

### Study site and subjects

We conducted this study in the Dindefelo Community Nature Reserve (hereafter Dindefelo), Kedougou region, south-east of Senegal. Dindefelo extends for over 140 km^2^ of savanna habitat (sensu Lindshield et al., 2021b) dominated by dry and deciduous vegetation types such as grassland, wooded grassland and woodland, with a small proportion of evergreen forest vegetation types, and some cropland areas (Dotras et al., 2024; Sánchez-Megías et al., 2024). The climate is highly seasonal with a five-month rainy season (June–October) and a seven-month dry season (November–May), a mean annual temperature of 28.5 °C, and a mean annual rainfall of 1,129 mm (Dotras et al., 2024; Sánchez-Megías et al., 2024). Dindefelo is home to a population of the Critically Endangered (Humle et al., 2016) western chimpanzee consisting of at least 53 adult individuals (Dotras et al., 2024; Sánchez-Megías et al., 2024) divided into two distinct communities (Segou and Nandoumary: Sánchez-Megías et al., 2026). Dindefelo chimpanzees are non-provisioned and non-habituated to human observers (Dotras et al., 2024).

### Data collection

We obtained 14 h 58 min of camera trap video footage at 14 locations in which we recorded Dindefelo chimpanzees performing tool-use behaviours (algae fishing, ant dipping and termite fishing; Figure 1). We set up camera traps (Bushnell Trophy Cam HD Aggressor Brown, Bushnell Trophy Cam HD Essential E3, Bushnell Core DS, Bushnell Core S-4 K, Browning BTC-7-4 K Edge, and Wimius H6) to record 1-min videos upon detecting movement and until it ceased, with a 0.5 or 1 s delay between consecutive videos. Between 2017 and 2024, we obtained 3 h 41 min of footage at five locations in which chimpanzees fished or attempted to fish for algae and recorded 33 successful and 46 unsuccessful algae fishing events. Between 2018 and 2022, we obtained 10 h 37 min of footage at four locations in which chimpanzees dipped for ants or looked for them inside their nests and recorded 166 successful and 525 unsuccessful ant dipping events. In 2018, 2019, 2022 and 2023, we obtained 40 min of footage at five locations in which chimpanzees fished for termites and recorded 105 successful termite fishing events.

**Figure 1:**
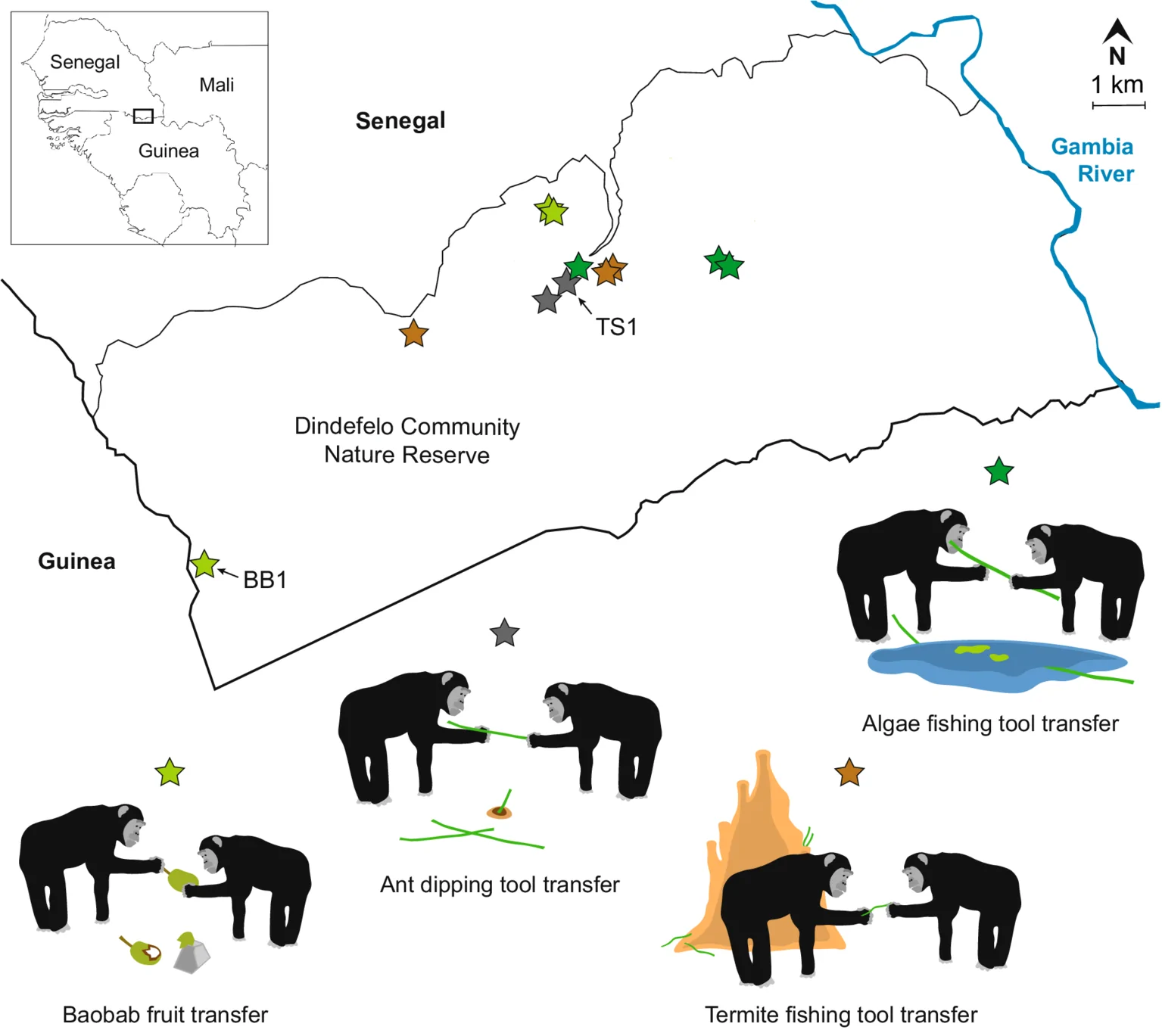
Map of Dindefelo in Senegal with the locations where we recorded algae-fishing (dark green), ant-dipping (dark grey) and termite-fishing (brown) tool transfers, and baobab fruit transfers (light green).

In total, we recorded 33 instances and three attempts of tool transfer, which we defined following Musgrave et al. (2016) as the change in possession of a tool between individuals. If a tool was transferred from one individual to another, and back to the initial possessor, we considered the second tool transfer to be non-independent and excluded it from the analyses (following Musgrave et al., 2016). For each tool transfer, and for initial possessor and recipient, we noted their age and sex, the time (s) they were visible before and after the transfer (up to 30 s), and the time (s) they used tools, the number of times they inserted a tool (into the water, ant nest or termite mound), and the number of times they ate (algae, ants or termites) before and after the transfer (following Musgrave et al., 2016). We did not include eating in unsuccessful ant dipping events, as ants were absent from the location and, thus, chimpanzees could not ingest them. We noted whether the initial possessor initiated tool use before the transfer.

Following Musgrave et al. (2020) we classified tool transfers as: proactive, when the initial possessor initiated the transfer; active, when the initial possessor divided a tool so a recipient could use a fragment or moved to facilitate the transfer; passive, when the recipient begged for the tool and grabbed it, and the initial possessor allowed it; tolerated take, when the recipient, without begging, took the tool from the initial possessor, who allowed it; hesitant, when the recipient begged for the tool and grabbed it, and the initial possessor resisted before relinquishing the tool; and steal, when the recipient forcibly took the tool from the initial possessor, who complained. We classified tool transfer attempts as: refusal, when the recipient begged for the tool but the initial possessor did not allow the transfer to occur; and failed steal, when a recipient tried to grab a tool, but the initial possessor complained and did not allow the transfer to occur (following Musgrave et al., 2020). We also classified as recovery those transfers in which the initial possessor left the tool and the recipient grabbed it whilst it was still within the initial possessor’s arm reach, following Pruetz and Lindshield (2012).

In addition, we obtained camera trap video footage at locations close to baobab trees where chimpanzees could potentially perform baobab pounding. We screened this footage looking for recordings in which multiple chimpanzees appeared and at least one was carrying baobab fruit, and therefore, baobab fruit transfer could potentially occur. We obtained 30 min of footage fulfilling these criteria at 11 locations (Figure 1) and recorded four instances in which chimpanzees transferred either whole baobab fruits or pieces of them. This footage was obtained in 2021, 2024 and 2025. Following Pruetz and Lindshield (2012), we classified baobab fruit transfers as: theft, when the recipient forcibly took the fruit from the initial possessor and the initial possessor complained; recovery, when the initial possessor abandoned the fruit and the recipient took it whilst it was still within the initial possessor’s arm reach; passive transfer, when the recipient took the fruit from the initial possessor and the initial possessor allowed it; active transfer, when the initial possessor divided the fruit and shared a portion with the recipient; and giving, when the initial possessor provided most or all of the fruit to the recipient.

Lastly, we described in detail one case of ant dipping tool transfer and one case of multiple baobab fruit transfers that hint at the social learning aspects involved in the transmission of technological behaviours.

### Data analysis

We conducted all statistical analyses in R v4.5.2 (R Core Team, 2025) using *α* = 0.05. We excluded from our analyses observations in which individuals were visible for <10s before or after the tool transfer, as we deemed these time frames to be too short to capture changes in chimpanzee behaviour. We used Linear Mixed-Effects Models (LMMs: function *lmer* in package *lme4* v1.1–38; Bates et al., 2015) to test whether initial tool possessors and tool recipients showed different behaviour (time using tools, number of tool insertions, and number of mouthfuls) after versus before the tool transfer. We excluded recoveries of abandoned tools from the models, given that in these cases the initial possessor did not interact with the recipient before voluntarily abandoning the tool. Since we included observations of different time durations (from 10 to 30 s), we standardised our variables per unit of time (s) and we also obtained standardised coefficients (function *standardise_parameters* in package *effectsize* v1.0.1; Ben-Shachar et al., 2020). We ran three different models with proportion of time using tools, insertions per second, and mouthfuls per second as response variables, respectively. We fitted each model twice, once for initial possessors and once for recipients. All models included a fixed effect for whether the observation corresponded to before or after the tool transfer (time frame) and a random effect for each individual tool transfer. To assess the significance of the models’ intercepts, we obtained their respective *p* values using Satterthwaite’s degrees of freedom method using the package *lmerTest* v3.2–0 (Kuznetsova et al., 2017). We fitted each model multiple times, each considering observations in which individuals were visible a minimum of 10 to 30 s in both time frames, to validate that selecting different inclusion criteria did not return different results. We checked that models satisfied assumptions and showed no strong deviations using the package *DHARMa* v0.5.0 (Hartig, 2026). AS-M analysed all the videos. AB validated individual sex and age and identified initial possessors and recipients whenever possible. KL-L analysed 20% of the videos selected at random and we confirmed that interobserver reliability was 85.7% for tool transfer types.

## Results

### Tool transfers

We recorded 33 tool transfers, the majority in the context of ant dipping (*n =* 19, 57.6%), followed by algae fishing (*n =* 10, 30.3%) and termite fishing (*n =* 4, 12.1%). In addition, we recorded two failed steals in which one individual tried to obtain an ant-dipping tool held by another individual, but the latter did not allow the transfer to occur. Most initial possessors were adults (*n =* 19, 57.6%), some were juveniles or adolescents (*n =* 9, 27.3%) and the rest were infants (*n =* 5, 15.2%). In contrast, most recipients were infants (*n =* 14, 42.4%), followed by adults (*n =* 10, 30.3%) and juveniles or adolescents (*n =* 9, 27.3%). Amongst initial possessors whose sex we could determine, most were females (*n =* 24, 80%) and only a few were males (*n =* 6, 20%), whereas all recipients of known sex were females (*n =* 24, 100%). Most transfers were from female adults to infants (either female: *n =* 6, 18.2%; or of unknown sex: *n =* 5, 15.2%), followed by female juveniles to female adults (*n =* 4, 12.1%), male adults to female adults (*n =* 4, 12.1%), and female infants to female juveniles (*n =* 3, 9.1%). We individually identified initial possessors and recipients in 27 (81.8%) transfers. Most transfers occurred between related individuals (*n =* 16, 59.3%): eight (29.6%) from mother to offspring, four (14.8%) from offspring to mother, and four (14.8%) between siblings. The rest involved unrelated individuals (*n =* 8, 29.6%) or individuals whose kinship is uncertain (*n =* 3, 11.1%).

Regarding tool transfer type, most transfers were recoveries in which a recipient took a tool that an initial possessor had just abandoned (*n =* 14, 42.4%), most frequently an infant recovering a tool left by a female adult (*n =* 6, 42.9% of recoveries). In tool recoveries, the initial possessor walked away from the tool and did not react to the recipient taking it. On three (9.1%) occasions, female adults recovered a fragment that had fallen from a tool which was being used by the initial possessor (two male adults and one female infant). In these cases, the initial possessor continued using a fragment of the initial tool and did not react to the recipient taking a fragment fallen from the tool. The second most frequent transfer type was tolerated take in which an initial possessor relinquished a tool that a recipient had grabbed (*n =* 13, 39.4%). We observed two (6.1%) steals in which an initial possessor complained about a recipient taking a tool, by moving their arms to hamper the transfer, unsuccessfully trying to hit the recipient or grab the tool back from them. We recorded one (3%) proactive transfer in which an initial possessor offered a tool to a recipient (described in Case 1). All transfers were initiated by the recipient except for the instance of proactive transfer (Case 1). In 24 (72.7%) cases, the initial possessors had already used or were using the tool at the moment of the transfer, whereas in nine (27.3%) cases, they had not started using the tool. We identified 13 individual initial possessors in 28 (84.8%) of the transfers and 14 individual recipients in 27 (81.8%) of the transfers. In addition, in two steal attempts we observed a tool possessor hitting or grabbing the tool from an individual trying to take it, successfully impeding the transfer.

Initial possessor and recipient behaviour significantly differed after versus before the tool transfer (not including recoveries). After transferring a tool, initial possessors used other tools for a lower proportion of time (minimum mean difference ± SD = −0.32 ± 0.59) and performed fewer insertions per second (−0.18 ± 0.27) compared to their tool use before the transfer (Table 1; see Supplementary Figure S1; Supplementary Tables S1, S3 for extended results). In contrast, recipients used tools for a higher proportion of time (0.56 ± 0.35), performed more insertions per second (0.24 ± 0.21), and ate more times per second (0.02 ± 0.04) after compared to before receiving a tool (Table 2; see Supplementary Figure S1; Supplementary Tables S2, S4 for extended results). These differences were significant in most of the models we tested.

**Table 1:** Results of LMMs fitted for initial possessors.

Response variable	Time visible	*N*	Std *β*	95% CI	SE	df	*t*	*p*
Proportion of time using tools	10	10	0.92	[0.06, 1.78]	0.17	18	2.27	0.036 *
	15	5	1.58	[0.82, 2.35]	0.14	4	5.06	0.007 **
	18	4	1.51	[0.44, 2.58]	0.19	3	3.91	0.03 *
Insertions per second	10	10	0.85	[−0.03, 1.73]	0.09	18	2.06	0.05
	15	5	1.50	[0.81, 2.18]	0.06	4	5.35	0.006 **
	18	4	1.39	[0.44, 2.33]	0.08	3	4.08	0.03 *
Mouthfuls per second	10	8	0.53	[−0.55, 1.62]	0.02	14	1.07	0.3
	15	5	1.12	[−0.19, 2.44]	0.02	4	2.09	0.11
	18	4	1.09	[−0.11, 2.29]	0.01	3	2.52	0.09

Each model was fitted considering observations in which individuals were visible a minimum of 10, 15 and 18 s before and after the transfer. Significance of *p* values: <0.001 ***, <0.01 **, <0.05 *.

**Table 2:** Results of LMMs fitted for recipients.

Response variable	Time visible	*N*	Std *β*	95% CI	SE	df	*t*	*p*
Proportion of time using tools	10	11	−1.60	[−2.13, −1.07]	0.1	20	−6.37	<0.001 ***
	15	8	−1.51	[−2.22, −0.80]	0.13	14	−4.65	<0.001 ***
	20	6	−1.73	[−2.33, −1.12]	0.11	6	−6.57	<0.001 ***
	24	4	−1.72	[−2.55, −0.90]	0.13	3.6	−5.83	0.006 **
Insertions per second	10	11	−1.50	[−2.09, −0.91]	0.04	20	−5.37	<0.001 ***
	15	8	−1.44	[−2.19, −0.68]	0.05	14	−4.15	<0.001 ***
	20	6	−1.63	[−2.36, −0.90]	0.06	6	−5.12	0.002 **
	24	4	−1.68	[−2.62, −0.74]	0.04	3.6	−4.95	0.01 *
Mouthfuls per second	10	9	−0.89	[−1.81, 0.04]	0.01	16	−2.05	0.06
	15	7	−0.81	[−1.93, 0.32]	0.01	12	−1.59	0.14
	20	5	−1.42	[−2.51, −0.33]	0.01	4.8	−3.19	0.03 *
	24	4	−1.69	[−2.60, −0.77]	0.01	3.6	−5.12	0.009 **

Each model was fitted considering observations in which individuals were visible a minimum of 10, 15, 20 and 24 s before and after the transfer. Significance of *p* values: <0.001 ***, <0.01 **, <0.05 *.

After transferring a tool, most of the initial possessors that were visible for at least 10 s stopped using tools (*n =* 13, 56.5%). Some initial possessors continued using tools by reusing one found on the ground (*n =* 5, 21.7%), manufacturing a new one (*n =* 3, 13%) or reusing the same one after the recipient stopped using it (*N =* 1, 4.3%). On an occasion (4.3%), the initial possessor divided the tool into two fragments and continued using one, whilst the recipient used the other one.

In addition, we recorded four baobab fruit transfers: in one (25%) case an initial possessor gave a whole unopened fruit to a recipient, and in the other three (75%) a recipient took a piece of a fruit that an initial possessor had cracked open. In two (50%) of these instances, female adults transferred the fruit to their male infants. The other two (50%) transfers involved individuals of uncertain kinship: a female adult letting a male juvenile take a fruit and a juvenile letting a male infant take and consume a piece of a fruit he had just opened. The transfer of the whole fruit and one fruit piece occurred during the same event (detailed in Case 2).

### Case 1: ant-dipping tool transfer

This case involved a female adult and a female infant who were ant dipping at an army ant nest (Supplementary Video S1). The female adult moved next to the infant and inserted the tool into the ant nest. She left the tool inserted into the nest, turned around to grab the infant, and brought the infant to the tool. Then, the infant grabbed the tool, retrieved it from the ant nest, and consumed the ants.

On February 3, 2018, at 1:26:03 p.m., we recorded a party of at least six chimpanzees composed of male adult Heewi, female adult Soukki with her female infant Saïsaï, female adult Hiila with her male infant Cheempo, and an unidentified female juvenile at ant nest location TS1 (Figure 1). Saïsaï was between 3.5 and 4 years old and Cheempo was about 1 month old on the date of the recording. Chimpanzees frequently visit this location and inspect the ant nest to see whether ants are present (Sánchez-Megías et al., 2024). Adults Heewi, Soukki and Hiila were ant dipping whilst standing on the ground around the ant nest entrance. Hiila was carrying Cheempo ventrally. The female juvenile was dipping for ants from farther away, standing on and holding with one hand onto a liana stem. At 1:28:49 p.m., infant Saïsaï started ant dipping.

At 1:31:43 p.m., whilst holding a tool with the left hand, Saïsaï grabbed a liana stem with both hands. She freed her left hand of the liana to continue dipping, whilst still holding onto the liana with her right hand. Soukki was dipping opposite of Saïsaï, with Heewi and Hiila between them, each on one side. Soukki inserted the tool into the ant nest, retrieved it and slid her right hand through it, which accumulated the ants. She quickly brought her right hand to the mouth and ate the ants. She repeated this sequence four times. She then walked bipedally around the ant nest, behind Hiila and towards Saïsaï, whilst holding the tool with her left hand and shaking ants off her right leg with her right hand. Saïsaï vocalised to Soukki and clung onto her belly whilst Soukki inserted the tool into the ant nest from the same place where Saïsaï was initially. Saïsaï let go of Soukki and stood next to her. Saïsaï started walking away from Soukki and behind Heewi. Soukki stopped inserting the tool, walked after Saïsaï, grabbed her arms and brought her back to the initial dipping position. Then, Saïsaï grabbed the tool that Soukki had left inserted into the ant nest with the left hand and foot, rapidly slid the right hand through the tool and brought it to the mouth to eat the ants. Soukki observed how Saïsaï performed this ant dip.

In the next recording, at 1:32:57 p.m., Saïsaï was walking bipedally whilst carrying in the left hand the tool that Soukki had given to her. Saïsaï switched the tool to her right hand and grabbed onto the same liana stem with her left hand. She switched the tool to her mouth and held onto the liana with her right hand. She let go of the liana with her left hand, switched the tool back to it, and continued dipping in this position. Soukki observed Saïsaï and the female juvenile, who had moved around the ant nest and behind Saïsaï. Then, at 1:33:32 p.m., Soukki walked out of the camera frame. Saïsaï continued dipping whilst standing on and holding onto liana stems until the end of this recording, at 1:33:54 p.m.

### Case 2: baobab fruit transfer and pounding facilitation

This case involved a female adult, a male juvenile and a male infant who were on a rocky outcrop (Supplementary Video S2). The female adult brought three baobab fruits to the outcrop. The infant playfully attempted to pound one fruit against the rocky surface whilst the female was holding it by the stem. The juvenile slapped and stamped the rock, then grabbed the fruit and moved it up and down whilst the infant was still holding it. Later, the juvenile cracked open the baobab fruit, and the infant consumed one piece from it. The infant grabbed another fruit and continued his pounding attempts.

On December 15, 2021, at 9:13:07 a.m., we recorded a party of at least six chimpanzees at location BB1 where we have recorded chimpanzees performing ant dipping and baobab pounding (Figure 1). The chimpanzees were female adult Féré with her male infant Fotti, male adult Bani, female juvenile Ârata, and two unidentified male juveniles. Bani, Ârata and Fotti were on a horizontal surface of a rocky outcrop. Chimpanzees are often seen in the outcrop, which emerges from a steep slope within a valley (Galán-Plana et al., 2026). This rock shelter includes a horizontal rock platform and a back wall which extends forward creating an overhung (Supplementary Video S2). Féré arrived at the outcrop transporting in her mouth three baobab fruits by their petioles and climbed onto the rock platform. With her left hand, she grasped the fruits and rested them on the ground without letting them go. Immediately, Fotti approached her, took one of the baobab fruits by the base of the petiole with the left hand, and swept the fruit along the rocky surface five times, slightly elevating it and hitting it against the rock platform but with insufficient strength so as to break it open. Féré was still holding the fruit by its long stem.

Seconds later, the male juvenile arrived and climbed onto the rock platform. Fotti left the fruit and chased the juvenile whilst making a pout face. The juvenile ran away from the infant whilst audibly slapping the rock platform nine times, with hands and feet. Fotti went back to grasp the baobab fruit, still held by Féré, turned back to look at the juvenile, and hit the fruit against the outcrop once more. The juvenile approached Fotti, who turned to face the juvenile and hit the fruit against the rock another five times, all whilst looking at the juvenile and displaying pout face. Whilst Fotti hit the fruit, the juvenile audibly stamped the outcrop four times in apparent synchrony with Fotti’s movements. The juvenile gripped the fruit, which Fotti was still holding by its petiole, and moved both the fruit and Fotti’s right hand in one baobab pounding motion. Whilst pouting, Fotti touched the juvenile’s hand, which released the fruit. Féré turned and moved her arm, apparently to intervene, but stopped halfway and scratched her head. The juvenile brought the right hand close to the fruit once more, but this time did not grab the fruit. Meanwhile, Fotti hit the fruit against the outcrop six more times, with wider swings than in previous attempts. The baobab fruit did not crack open. Whilst observing Fotti’s attempts, the juvenile audibly stamped the rock thrice more. Fotti grabbed the fruit again and hit it against the flat surface four more times. Féré allowed Fotti to grab the fruit by letting it go of her right hand and foot and transferring it to Fotti with her left hand.

In the following recording, at 9:14:33 a.m., the juvenile was holding one baobab fruit and hit it five times against the rocky platform before swinging it back and forth towards Féré and Fotti. Féré moved her arm to prevent the juvenile from potentially clubbing them. After hearing the pounding sounds, Fotti approached, and the juvenile swung the fruit once more against the outcrop right beside Fotti. Then, Fotti sat down and attentively observed the juvenile’s movements. The juvenile pounded the fruit five times eventually cracking it. With both hands and the mouth, the juvenile opened the cracked fruit and began consuming its pulp. Fotti collected a scattered fruit piece from the outcrop that had broken off and ate it. Then, Fotti went back to Féré and grasped the second baobab fruit, which Féré had left on the outcrop and was unopened. Féré was eating the third fruit, which was already opened when she entered the camera frame in the first recording. The juvenile transported the first fruit with the right hand, descending the outcrop feet first and climbing it again behind Féré and away from Fotti. Fotti moved his free hand and pulled the second fruit he was holding away from the juvenile, seemingly to prevent the juvenile from taking it. Fotti dragged this second baobab fruit with the left hand, sat in the same position where the juvenile had cracked the first fruit, and unsuccessfully attempted to elevate the second fruit from the outcrop three more times in a pounding motion before successfully hitting it once, stopping, and biting the distal end of its petiole.

In the last recording, at 9:15:43 a.m., Fotti dragged the fruit along the surface of the outcrop, but the fruit fell off the outcrop onto the ground below. Fotti approached the edge of the outcrop and looked at the fallen fruit. Finally, Fotti descended from the platform feet first and moved outside of the camera frame.

## Discussion

The majority of tool transfers we observed occurred from skilled to inexperienced individuals. Excluding recoveries, transfers of this kind fulfil the criteria to be considered teaching proposed by Caro and Hauser (1992). Our models show that tool transfers represented a cost for the initial possessors, who exhibited significantly less tool-use behaviour after the transfer, and on most occasions stopped using tools. Initial possessors did not significantly decrease their mouthfuls per second after a transfer. When analysing this we excluded events in which there was no food for the chimpanzees to eat, so this result could be due to the small sample sizes. At the same time, tool transfers supposed a benefit for the recipients, who engaged in significantly more tool-use and feeding behaviour. These concur with previous findings supporting that tool transfers are a form of teaching in chimpanzees (Musgrave et al., 2016).

Besides recoveries of abandoned tools, tolerated takes were the most frequent transfer type in Dindefelo. Similarly, the most common tool transfer types at Fongoli, Senegal, were passive transfers (Pruetz and Lindshield, 2012). Passive transfers (Pruetz and Lindshield, 2012) are equivalent to tolerated takes (Musgrave et al., 2020). We did not observe any active transfer, although we recorded the proactive transfer described in Case 1. In the context of termite fishing, Musgrave et al. (2020) found that tolerated takes were the most common transfer type in Gombe (Tanzania), whereas active transfers were the most common transfer type in Goualougo, and hypothesised that this difference responded to the varying complexity of termite-fishing strategies between these two sites. They proposed that acquiring termite-fishing skills may demand more facilitation in Goualougo than in Gombe, given that Goualougo chimpanzees modify tools to create brush-like tips, use thick perforating sticks to open holes in termite mounds before fishing, and select specific raw material to make their tools, which does not occur in Gombe. That study focused on tool transfers from mother to offspring, highlighting the role of mothers in teaching termite-fishing skills to their young. Here, we studied transfers from experienced to naïve individuals. The case of Dindefelo more closely resembles that of Gombe, since the tool-use behaviours we studied do not seem to involve the use of tool sets (Boesch et al., 2020; Sánchez-Megías et al., 2026) and tolerated takes were more common than active transfers in this site. Our findings support the idea that there may be more facilitation involved in the transmission of technological behaviours in sites where these behaviours are more complex, as Musgrave et al. (2020) hypothesised for termite fishing.

In addition, donors in Goualougo were more likely to transfer tools to requesting immature recipients than donors in Gombe, which were more likely to resist a transfer, supporting the idea that donors in Goualougo participate more actively in the transmission of termite-fishing skills than in Gombe (Musgrave et al., 2020). In Dindefelo, most recipients were met with no resistance from initial tool possessors, but further research is needed to assess whether specific tool transfer types occur in different frequencies than in Gombe or Goualougo. Chimpanzees in savanna habitats, like those in Dindefelo, exhibit high levels of social cohesion and tolerance (Lindshield et al., 2021b). In Fongoli, chimpanzees display high tolerance to food transfers and displacement by other individuals (Pruetz and Lindshield, 2012). It may be that the high tolerance savanna chimpanzees display in these contexts also translates into high tolerance to tool transfers.

Noteworthily, we found that most initial tool possessors and all tool recipients in Dindefelo whose sex we could determine were females. In Fongoli, most transfers occurred from males to females, suggesting that males there have a high tolerance to sharing resources with subordinates (Pruetz and Lindshield, 2012). In that site, all tool recipients were also females, although few cases were reported (Pruetz and Lindshield, 2012). In both Gombe and Goualougo, females were significantly more likely to be given tools compared to males, regardless of whether the tools had been requested (Musgrave et al., 2020). Since recipients of both sexes requested tools in similar frequencies in Gombe and Goualougo, there appeared to be different reactions of mothers towards male and female infant requests (Musgrave et al., 2020). It seems that in Dindefelo young females may also be more likely to receive tools than young males. Further research is needed to confirm whether this sex difference in the transmission of tool-use skills exists in Dindefelo. The fact that tool requests elicit distinct reactions from mothers depending on the sex of their infant may contribute to establishing the sex differences in extractive tool-use exhibited by older individuals. These differences include the faster termite-fishing acquisition by female compared to male juveniles in Gombe (Lonsdorf et al., 2004), the higher nut-cracking efficiency in female compared to male adults in Taï (Côte d’Ivoire: Boesch and Boesch, 1981) and the higher engagement in ant fishing in female compared to male adults in Mahale (Tanzania: Hiraiwa-Hasegawa, 1989).

Additionally, Case 1 and Case 2 demonstrate that other social mechanisms may contribute to the transmission of technological behaviours. In Case 1, the mother actively modified her behaviour to facilitate her infant’s tool use, as she stopped ant dipping, left the tool inserted, and brought her infant to her tool, so that the infant would retrieve it from the ant nest and consume the ants. This supports previous findings that chimpanzees can proactively transfer tools, and thus may detect subtle signs that an individual is in need of assistance (Musgrave et al., 2020). In Case 2, the juvenile also altered his behaviour in the presence of the infant, which initially produced no benefit to the juvenile and which seemed directed towards encouraging the infant’s baobab-pounding attempts, which fulfil criteria for teaching (Caro and Hauser, 1992; Hoppitt et al., 2008). However, in this case there was an overall benefit for the juvenile, as he ended up getting one fruit and consuming it. Case 2 fulfils additional criteria for teaching (sensu Musgrave et al., 2016), as the juvenile produced ostensive signals, both visual and auditory (slapping and stamping the rock), and shifted from ostensive signalling to accompanying the infant’s behaviour and to demonstrating a successful baobab-pounding attempt, which may reflect his understanding of the infant’s level of ability.

In Case 2, the mother transported fruits to the outcrop and facilitated the interaction by allowing the infant and the juvenile to manipulate and take the fruits. Similarly to tool transfers, food transfers facilitate learning foraging strategies by providing adequate resources to naïve individuals (Boesch, 1991; Boesch and Boesch-Achermann, 2000). Baobab fruit transfers have been reported in Fongoli, and most were passive transfers (Pruetz and Lindshield, 2012). Similarly, we found that in the few baobab fruit transfers we observed in Dindefelo, the initial possessor tolerated the recipient taking a whole fruit or a piece. In Case 2, the mother provided the youngsters with the necessary materials (baobab fruit in the presence of an anvil) for them to practise baobab pounding, similar to the creation of discovery environments observed in humans (sensu Whiten, 1999). Our findings show that, albeit less complex than tool-use behaviours, proto-tool use behaviours may also require facilitation and teaching to be acquired by immature chimpanzees.

Case 2 also hints at the role of juveniles in skill transmission. The juvenile assisted (sensu Whiten, 1999) the infant in pounding the fruit. Chimpanzees slap and stamp rocks to invite and entice infant play (Nishida et al., 2010), so the juvenile may have been encouraging the infant’s attempts. Moreover, it is possible that slapping and stamping the rocky surface may have provided cues about the pounding action, both visual (the hitting movement) and auditory (the hitting sound). Chimpanzees and other primates spontaneously synchronise their movements to those of others and to visual and auditory cues (Fuhrmann et al., 2014; Yu et al., 2018; Hattori and Tomonaga, 2020). Their mirror neuron system integrates motor, visual, and auditory representations of movements (Kohler et al., 2002; Sugihara et al., 2006; Le Bel et al., 2009; Huang et al., 2019; Huang and Brosch, 2020). The juvenile’s use of ostensive visual and auditory signals resembling pounding movements could have aided the infant understand the pounding action and focused his attention on the effect of the movement. However, although the juvenile’s actions may have benefitted the infant by providing an opportunity to observe a successful pounding event, we cannot dismiss that the juvenile was simply reacting to Fotti’s movements or that he aimed to steal the fruit.

After that, the juvenile grabbed the fruit and performed a pounding movement whilst the infant was holding it by the pericarp, thus guiding the infant’s movement. Nonetheless, we cannot discount that the juvenile unsuccessfully tried to steal the fruit from the infant. Later, the juvenile appeared holding the same fruit that the infant was initially holding, given that the adult female was still holding the other two fruits. However, how he obtained it was not recorded, he may have stolen it from the infant. Moments later, the juvenile pounded the fruit until one fragment visibly detached and was ejected, indicating that the fruit was almost cracked open. The juvenile stopped, looked at the infant, pounded the fruit close to the infant, and then swung it seemingly to club the infant. Clubbing others is another type of play invitation (sensu Nishida et al., 2010) which the juvenile could have used to catch the infant’s attention. When the infant had approached and was observing, the juvenile demonstrated (sensu Whiten, 1999) a successful baobab opening and started consuming the pulp. The infant collected a scattered piece and ate it. Letting learners consume part of the food familiarises them with it (Boesch, 1991) and may act as a reward that motivates them to keep trying. Ensuing, the juvenile left, concluding the learning event. After this interaction, the infant returned to the mother to get another fruit and continued attempting to break it open.

Chimpanzee mothers are known to adapt their nut-cracking teaching strategies depending on their infant’s understanding to tackle problematic points (Boesch and Boesch-Achermann, 2000). Case 2 suggests that juveniles may also be able to perceive the ability and understanding of others and adapt their behaviour accordingly, offering an insight into the ontogeny of teaching abilities in chimpanzees. Infants mostly learn from adults, especially their mothers (Boesch, 1991; Humle et al., 2009; Musgrave et al., 2016). Adults are more skilled than juveniles (Boesch, 1991; Humle et al., 2009) and may represent better observational models. Since chimpanzees are known to identify and copy more knowledgeable conspecifics (Matsuzawa et al., 2001; Kendal et al., 2015), and juveniles are more experienced than infants, the former can act as models for the latter. Older siblings may act as observational models alongside mothers from whom infants may acquire new skills. Like mothers, older siblings share long periods of time with infants, providing more learning opportunities than unrelated individuals. This social closeness facilitates that the naïve individual identifies with the experienced one (de Waal and Bonnie, 2009). Additionally, older siblings are also to benefit from their siblings’ learning, since it increases their fitness, as suggested for mothers (Musgrave et al., 2016). However, infants at Bossou (Guinea) were less likely to observe nut cracking by their siblings compared to their mothers or unrelated conspecifics (Biro et al., 2003). Our findings suggest that juveniles can act as observational models of technological skills for infants, but more data are needed to understand the role of siblings in technological behaviour acquisition.

## Conclusion

Our findings on algae-fishing, ant-dipping and termite-fishing tool transfers in Dindefelo support our hypothesis that these actions represent a type of teaching that contributes to the transmission of tool-use skills in wild chimpanzees, as it has been demonstrated for termite fishing in Goualougo (Musgrave et al., 2016). It appears that most tool-use social learning opportunities are created by the recipient’s initiative to grab a tool, which the initial possessor tolerates or facilitates by leaving tools behind. However, our results indicate that experienced individuals sometimes actively facilitate tool use. In Gombe and Goualougo, female offspring were more likely to receive termite-fishing tools upon request than male offspring (Musgrave et al., 2020). Our results suggest that females may receive tools more frequently than males in Dindefelo. In addition, our results suggest that facilitation, mainly from female adults but also from juveniles, contributes to the acquisition of technological behaviours in infant chimpanzees. This expands our knowledge on the facilitation of infant learning by experienced individuals, especially mothers (Boesch, 1991; Boesch and Boesch-Achermann, 2000; Humle et al., 2009), and raises questions regarding the role of juveniles as models, as well as the effect of being instructed by individuals with various skill levels. Our findings also show that the acquisition of proto-tool use behaviours, such as baobab pounding, may also be mediated by facilitation and teaching. More data from Dindefelo are needed to test whether chimpanzees there exhibit different teaching and facilitation patterns compared to elsewhere. Similarly, further research in other sites will allow us to make comparisons and understand the role of teaching and facilitation across different habitats, chimpanzee communities, and technological behaviours of varying degrees of complexity.

## Data Availability

The original contributions presented in the study are included in the article/Supplementary material, further inquiries can be directed to the corresponding authors.
