# Supplementary material for: Savanna chimpanzee tool and fruit transfers facilitate the transmission of technological behaviours at Dindefelo, Senegal
Source: Front Psychol. 2026 Sep 16;17:1868894. doi: 10.3389/fpsyg.2026.1868894 (PMC13624102; doi:10.3389/fpsyg.2026.1868894)
Supplement: Supplementary file 1 [file Data_Sheet_1.PDF]

Supplementary Information for:

Savanna chimpanzee tool and fruit transfers facilitate the transmission of technological behaviours at Dindefelo, Senegal

Authors

Andreu Sánchez-Megías[1,2], Amanda Barciela[2], Kevin López-Leal[2], Flavia Piccolella[2], Carlota F. Galán-Plana[3], Nadia Mirghani[2], Manuel Llana[2], Jordi Galbany[2,4,5], Laia Dotras[1,2], Justinn Renelies-Hamilton[2], Adrián Arroyo[6,7] and R. Adriana Hernandez-Aguilar[1,2,8]

Author's affiliations

[1]Department of Social Psychology and Quantitative Psychology, Faculty of Psychology, University of Barcelona, Barcelona, Spain.
[2]Jane Goodall Institute Spain in Senegal, Dindefelo Biological Station, Dindefelo, Kedougou, Senegal.
[3]Department of Primate Behavior and Evolution, Max Planck Institute for Evolutionary Anthropology, Leipzig, Germany.
[4]Department of Clinical Psychology and Psychobiology, Faculty of Psychology, University of Barcelona, Barcelona, Spain.
[5]Institute of Neurosciences, University of Barcelona, Barcelona, Spain.
[6]Seminar on Prehistoric Studies and Research (SERP), University of Barcelona, Barcelona, Spain.
[7]Institute of Archaeology of the University of Barcelona (IAUB), Barcelona, Spain.
[8]Serra Hunter Programme, Generalitat de Catalunya, Spain.

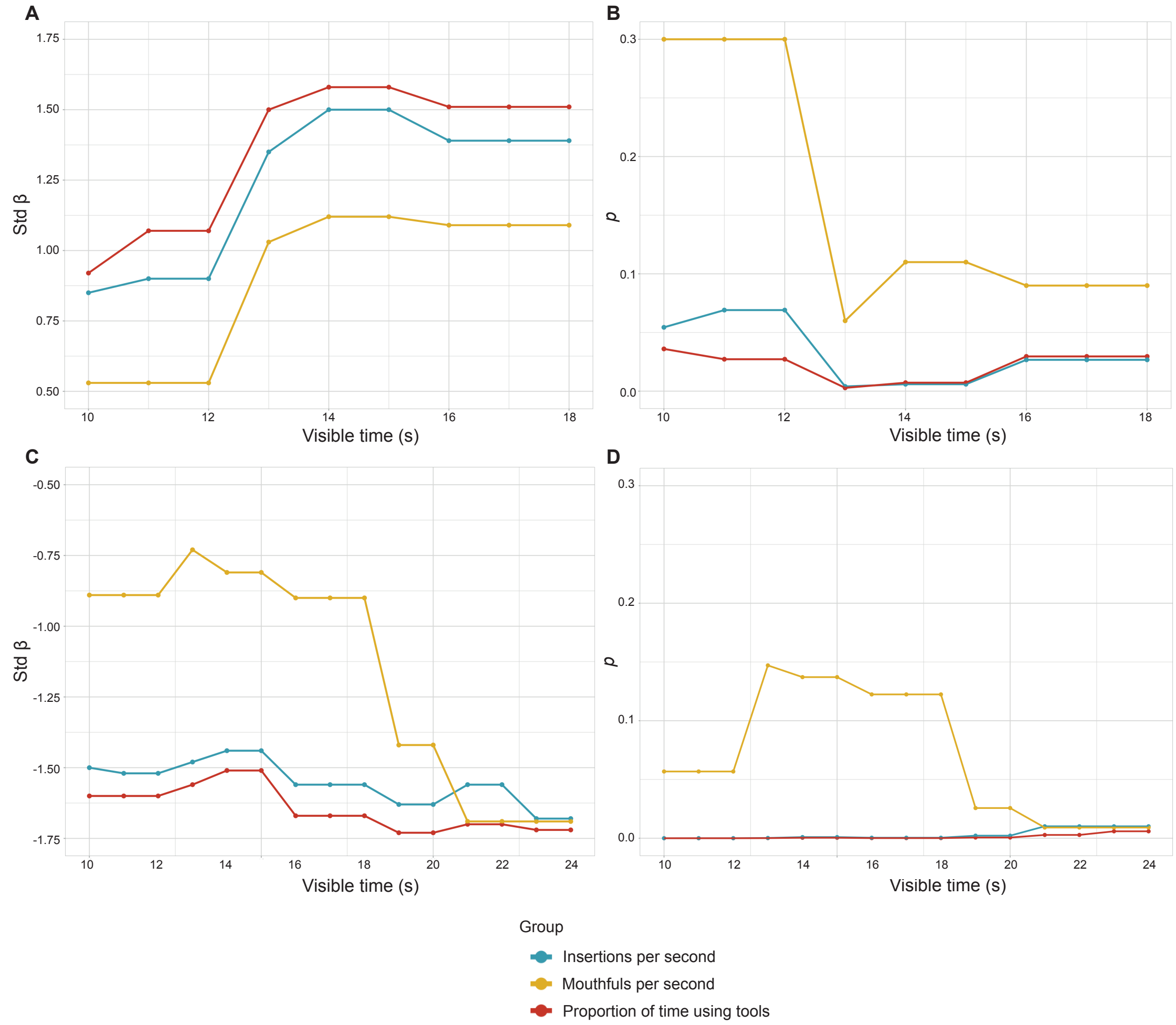

Figure S1. Changes in chimpanzee behaviour after versus before a tool transfer. Stacked line graphs show standardised coefficients and *p* values (y-axis) of Linear Mixed-Effects Models (LMMs) fitted multiple times, each considering observations in which individuals were visible a minimum of 10 to 18s (initial possessors) or 24s (recipients) before and after the transfer (x-axis). Models had time frame (before or after the transfer) as fixed effect, individual tool transfer as random effect, and proportion of time using tools, insertions per second, and mouthfuls per second as response variables, respectively. Cases with insufficient observations to fit an LMM were excluded. Variation in (**A**) standardised coefficients for the effect of time frame in initial tool possessor behaviour and (**B**) their *p* values, and in (**C**) standardised coefficients for the effect of time frame in tool recipient behaviour and (**D**) their *p* values.

Table S1. Results of LMMs fitted for initial possessors with proportion of time using tools, insertions per second, and mouthfuls per second as response variables, time frame (before or after the transfer) as fixed effect, and individual tool transfer as random effect. Each model was fitted considering observations in which individuals were visible a minimum of 10 to 18s before and after the transfer. Cases with minimum time visible between 19 and 30s were excluded due to insufficient observations to fit an LMM. Significance of *p* values: <0.001 ***, <0.01 **, <0.05 *.

Response variable	Time visible	*N*	Std β	95% CI	SE	df	t	*p*
Proportion of time using tools	10	10	0.92	[0.06, 1.78]	0.17	18	2.27	0.036 *
	11	8	1.07	[0.12, 2.01]	0.2	14	2.47	0.027 *
	12	8	1.07	[0.12, 2.01]	0.2	14	2.47	0.027 *
	13	7	1.50	[0.81, 2.19]	0.14	6	4.86	0.003 **
	14	5	1.58	[0.82, 2.35]	0.14	4	5.06	0.007 **
	15	5	1.58	[0.82, 2.35]	0.14	4	5.06	0.007 **
	16	4	1.51	[0.44, 2.58]	0.19	3	3.91	0.03 *
	17	4	1.51	[0.44, 2.58]	0.19	3	3.91	0.03 *
	18	4	1.51	[0.44, 2.58]	0.19	3	3.91	0.03 *
Insertions per second	10	10	0.85	[-0.03, 1.73]	0.09	18	2.06	0.05
	11	8	0.90	[-0.10, 1.90]	0.09	14	1.97	0.07
	12	8	0.90	[-0.10, 1.90]	0.09	14	1.97	0.07
	13	7	1.35	[0.68, 2.01]	0.06	6	4.52	0.004 **
	14	5	1.50	[0.81, 2.18]	0.06	4	5.35	0.006 **
	15	5	1.50	[0.81, 2.18]	0.06	4	5.35	0.006 **
	16	4	1.39	[0.44, 2.33]	0.08	3	4.08	0.03 *
	17	4	1.39	[0.44, 2.33]	0.08	3	4.08	0.03 *
	18	4	1.39	[0.44, 2.33]	0.08	3	4.08	0.03 *
Mouthfuls per second	10	8	0.53	[-0.55, 1.62]	0.02	14	1.07	0.3
	11	8	0.53	[-0.55, 1.62]	0.02	14	1.07	0.3
	12	8	0.53	[-0.55, 1.62]	0.02	14	1.07	0.3
	13	7	1.03	[0.02, 2.05]	0.02	6	2.26	0.06
	14	5	1.12	[-0.19, 2.44]	0.02	4	2.09	0.11
	15	5	1.12	[-0.19, 2.44]	0.02	4	2.09	0.11
	16	4	1.09	[-0.11, 2.29]	0.01	3	2.52	0.09
	17	4	1.09	[-0.11, 2.29]	0.01	3	2.52	0.09
	18	4	1.09	[-0.11, 2.29]	0.01	3	2.52	0.09

Table S2. Results of LMMs fitted for recipients with proportion of time using tools, insertions per second, and mouthfuls per second as response variables, time frame (before or after the transfer) as fixed effect, and individual tool transfer as random effect. Each model was fitted considering observations in which individuals were visible a minimum of 10 to 24s before and after the transfer. Cases with minimum time visible between 25 and 30s were excluded due to insufficient observations to fit an LMM. Significance of *p* values: <0.001 ***, <0.01 **, <0.05 *.

Response variable	Time visible	*N*	Std β	95% CI	SE	df	t	*p*
Proportion of time using tools	10	11	-1.60	[-2.13, -1.07]	0.1	20	-6.37	<0.001 ***
	11	10	-1.60	[-2.16, -1.04]	0.1	18	-6.06	<0.001 ***
	12	10	-1.60	[-2.16, -1.04]	0.1	18	-6.06	<0.001 ***
	13	9	-1.56	[-2.18, -0.93]	0.11	16	-5.36	<0.001 ***
	14	8	-1.51	[-2.22, -0.80]	0.13	14	-4.65	<0.001 ***
	15	8	-1.51	[-2.22, -0.80]	0.13	14	-4.65	<0.001 ***
	16	7	-1.67	[-2.29, -1.06]	0.11	12	-6.07	<0.001 ***
	17	7	-1.67	[-2.29, -1.06]	0.11	12	-6.07	<0.001 ***
	18	7	-1.67	[-2.29, -1.06]	0.11	12	-6.07	<0.001 ***
	19	6	-1.73	[-2.33, -1.12]	0.11	6	-6.57	<0.001 ***
	20	6	-1.73	[-2.33, -1.12]	0.11	6	-6.57	<0.001 ***
	21	5	-1.70	[-2.43, -0.96]	0.12	4.8	-5.62	0.003 **
	22	5	-1.70	[-2.43, -0.96]	0.12	4.8	-5.62	0.003 **
	23	4	-1.72	[-2.55, -0.90]	0.13	3.6	-5.83	0.006 **
	24	4	-1.72	[-2.55, -0.90]	0.13	3.6	-5.83	0.006 **
Insertions per second	10	11	-1.50	[-2.09, -0.91]	0.04	20	-5.37	<0.001 ***
	11	10	-1.52	[-2.13, -0.90]	0.05	18	-5.24	<0.001 ***
	12	10	-1.52	[-2.13, -0.90]	0.05	18	-5.24	<0.001 ***
	13	9	-1.48	[-2.16, -0.81]	0.05	16	-4.72	<0.001 ***
	14	8	-1.44	[-2.19, -0.68]	0.05	14	-4.15	<0.001 ***
	15	8	-1.44	[-2.19, -0.68]	0.05	14	-4.15	<0.001 ***
	16	7	-1.56	[-2.29, -0.84]	0.05	12	-4.81	<0.001 ***
	17	7	-1.56	[-2.29, -0.84]	0.05	12	-4.81	<0.001 ***
	18	7	-1.56	[-2.29, -0.84]	0.05	12	-4.81	<0.001 ***
	19	6	-1.63	[-2.36, -0.90]	0.06	6	-5.12	0.002 **
	20	6	-1.63	[-2.36, -0.90]	0.06	6	-5.12	0.002 **
	21	5	-1.56	[-2.49, -0.63]	0.07	4.8	-4.1	0.01 *
	22	5	-1.56	[-2.49, -0.63]	0.07	4.8	-4.1	0.01 *
	23	4	-1.68	[-2.62, -0.74]	0.04	3.6	-4.95	0.01 *
	24	4	-1.68	[-2.62, -0.74]	0.04	3.6	-4.95	0.01 *
Mouthfuls per second	10	9	-0.89	[-1.81, 0.04]	0.01	16	-2.05	0.06
	11	9	-0.89	[-1.81, 0.04]	0.01	16	-2.05	0.06
	12	9	-0.89	[-1.81, 0.04]	0.01	16	-2.05	0.06
	13	8	-0.73	[-1.78, 0.31]	0.01	14	-1.53	0.15
	14	7	-0.81	[-1.93, 0.32]	0.01	12	-1.59	0.14
	15	7	-0.81	[-1.93, 0.32]	0.01	12	-1.59	0.14
	16	6	-0.90	[-2.13, 0.33]	0.02	10	-1.69	0.12
	17	6	-0.90	[-2.13, 0.33]	0.02	10	-1.69	0.12
	18	6	-0.90	[-2.13, 0.33]	0.02	10	-1.69	0.12
	19	5	-1.42	[-2.51, -0.33]	0.01	4.8	-3.19	0.03 *
	20	5	-1.42	[-2.51, -0.33]	0.01	4.8	-3.19	0.03 *
	21	4	-1.69	[-2.60, -0.77]	0.01	3.6	-5.12	0.009 **
	22	4	-1.69	[-2.60, -0.77]	0.01	3.6	-5.12	0.009 **
	23	4	-1.69	[-2.60, -0.77]	0.01	3.6	-5.12	0.009 **
	24	4	-1.69	[-2.60, -0.77]	0.01	3.6	-5.12	0.009 **

Table S3. Changes in initial tool possessor behaviour after versus before a tool transfer. Mean differences in proportion of time using tools, insertions per second, and mouthfuls per second exhibited by initial tool possessors after compared to before transferring a tool. Each case was calculated considering observations in which initial possessors were visible a minimum of 10 to 18s before and after the transfer. Cases with minimum time visible between 19 and 30s were excluded due to insufficient observations to fit an LMM.

Time visible (s)	Proportion of time using tools			Insertions per second			Mouthfuls per second		
	N	Mean	SD	*N*	Mean	SD	*N*	Mean	SD
10	10	-0.39	0.65	10	-0.18	0.31	8	-0.02	0.06
11	8	-0.48	0.66	8	-0.19	0.29	8	-0.02	0.06
12	8	-0.48	0.66	8	-0.19	0.29	8	-0.02	0.06
13	7	-0.68	0.37	7	-0.28	0.16	7	-0.04	0.05
14	5	-0.73	0.32	5	-0.34	0.14	5	-0.05	0.05
15	5	-0.73	0.32	5	-0.34	0.14	5	-0.05	0.05
16	4	-0.73	0.37	4	-0.32	0.16	4	-0.03	0.02
17	4	-0.73	0.37	4	-0.32	0.16	4	-0.03	0.02
18	4	-0.73	0.37	4	-0.32	0.16	4	-0.03	0.02

Table S4. Changes in tool recipient behaviour after versus before a tool transfer. Mean differences in proportion of time using tools, insertions per second, and mouthfuls per second exhibited by tool recipients after compared to before receiving a tool. Each case was calculated considering observations in which recipients were visible a minimum of 10 to 24s before and after the transfer. Cases with minimum time visible between 25 and 30s were excluded due to insufficient observations to fit an LMM.

Time visible (s)	Proportion of time using tools			Insertions per second			Mouthfuls per second		
	N	Mean	SD	*N*	Mean	SD	*N*	Mean	SD
10	11	0.61	0.33	11	0.24	0.15	9	0.03	0.05
11	10	0.63	0.34	10	0.25	0.16	9	0.03	0.05
12	10	0.63	0.34	10	0.25	0.16	9	0.03	0.05
13	9	0.6	0.35	9	0.23	0.15	8	0.02	0.04
14	8	0.58	0.37	8	0.22	0.16	7	0.02	0.05
15	8	0.58	0.37	8	0.22	0.16	7	0.02	0.05
16	7	0.66	0.32	7	0.25	0.15	6	0.03	0.05
17	7	0.66	0.32	7	0.25	0.15	6	0.03	0.05
18	7	0.66	0.32	7	0.25	0.15	6	0.03	0.05
19	6	0.74	0.28	6	0.28	0.14	5	0.04	0.03
20	6	0.74	0.28	6	0.28	0.14	5	0.04	0.03
21	5	0.69	0.27	5	0.27	0.15	4	0.06	0.02
22	5	0.69	0.27	5	0.27	0.15	4	0.06	0.02
23	4	0.76	0.26	4	0.21	0.09	4	0.06	0.02
24	4	0.76	0.26	4	0.21	0.09	4	0.06	0.02
